# Mitochondrial calpain-1 activates NLRP3 inflammasome by cleaving ATP5A1 and inducing mitochondrial ROS in CVB3-induced myocarditis

**DOI:** 10.1007/s00395-022-00948-1

**Published:** 2022-08-23

**Authors:** Xiaoxiao Liu, Minghui Li, Zhiwei Chen, Yong Yu, Hui Shi, Ying Yu, Yucheng Wang, Ruizhen Chen, Junbo Ge

**Affiliations:** Department of Cardiology, Shanghai Institute of Cardiovascular Diseases, Zhongshan Hospital, Shanghai Medical College of Fudan University, Shanghai, China

**Keywords:** Calpain, NLRP3 inflammasome, Mitochondrial ROS, Viral myocarditis, ATP5A1

## Abstract

Treatment options for myocarditis are currently limited. Inhibition of calpains has been shown to prevent Coxsackievirus B3 (CVB3)-induced cardiac injuries, but the underlying mechanism of action of calpains has not been elucidated. We investigated whether NOD-, LRR-, and pyrin domain-containing 3 (NLRP3) inflammasome participated in CVB3-induced myocarditis, and investigated the effects of calpain-1 on CVB3-induced cardiac injury. NLRP3 inflammasome was activated in CVB3-infected hearts, evidenced by elevated protein levels of NLRP3, N-terminal domain of Gasdermin D, and cleaved caspase-1, and the increased co-localization of NLRP3 and apoptosis-associated speck-like protein. The intraperitoneal administration of MCC950, a selective inhibitor of the NLRP3 inflammasome, led to decreased levels of serum creatine kinase-MB, cardiac troponin I, lactate dehydrogenase, interleukin-18, interleukin-1β, prevention of the infiltration of inflammatory cells, and improvement of cardiac function under CVB3 infection. Transgenic mice overexpressing the endogenous calpain inhibitor calpastatin (Tg-CAST mice) exhibited not only decreased apoptosis, inflammation, fibrosis, and enhanced cardiac function but also inhibition of NLRP3 inflammasome and pyroptosis. The selective inhibition of calpain-1 using PD151746 protected cardiomyocytes in vitro from CVB3 infection by downregulating NLRP3 inflammasome and, thus, preserved cell viability. Mechanistically, we showed that mitochondrial dysfunction preceded inflammatory response after CVB3 treatment and elimination of mitochondrial reactive oxygen species (ROS) using mitochondria-targeted antioxidants (mito-TEMPO) recapitalized the phenotype observed in Tg-CAST mice. Furthermore, the promotion or inhibition of calpain-1 activation in vitro regulated the mitochondrial respiration chain. Mito-TEMPO reversed calpain-1-mediated NLRP3 inflammation activation and cell death. We also found that mitochondrial calpain-1, which was increased after CVB3 stimulation, activated the NLRP3 inflammasome and resulted in cell death. Furthermore, ATP synthase-α (ATP5A1) was revealed to be the cleaving target of calpain-1 after CVB3 treatment. Downregulating ATP5A1 using ATP5A1-small interfering RNA impaired mitochondrial function, decreased cell viability, and induced NLRP3 inflammasome activation. In conclusion, CVB3 infection induced calpain-1 accumulation in mitochondria, and led to subsequent ATP5A1 cleavage, mitochondrial ROS overproduction, and impaired mitochondrial function, eventually causing NLRP3 inflammasome activation and inducing pyroptosis. Therefore, our findings established the role of calpain in viral myocarditis and unveiled its underlying mechanism of its action. Calpain appears as a promising target for the treatment of viral myocarditis.

## Introduction

Myocarditis is an inflammatory disease of the heart frequently resulting from viral infections and/or post-viral immune-mediated responses [52]. Although most individuals affected with myocarditis recover, up to 20% of these patients develop chronic myocarditis, leading to dilated cardiomyopathy (DCM) and congestive heart failure [13]. Myocarditis has been identified as the third leading cause (6%) of cardiovascular deaths in young athletes, next only to coronary artery abnormalities (17%) and hypertrophic cardiomyopathy (36%) [34, 50]. Although remarkable progress in the diagnosis, pathophysiological mechanisms, and treatment of acute myocarditis have been made in the last few years, there exist no standard treatment strategies for acute myocarditis besides standard heart failure therapy and physical rest. Myocarditis is often triggered by infections caused by common viruses, mostly enteroviruses like Coxsackievirus B3 (CVB3), that have a predilection to entry into the myocardium. The pathophysiology of myocarditis in humans is not completely understood, but animal models of viral myocarditis predict a maladaptive post-viral immune-mediated response, which leads to eventual myocardial cell dysfunction and compromised contractility [22]. Immunosuppressive and immunomodulatory therapies have been shown to be beneficial in several randomized controlled trials [42], however, the immune mechanisms underlying the development of viral myocarditis need to be further investigated.

Pyroptosis is a form of necrotic and inflammatory programmed cell death, initiated by the canonical caspase-1-dependent and noncanonical caspase-4/5/11-mediated (human caspase-4/5 and murine caspase-11) pyroptosis pathways [59], of which the canonical pathways are usually activated within an inflammasome. An inflammasome is a macromolecular protein complex composed of inflammasome-initiating sensors (NLRP1, NLRP3, NLRC4, AIM2, or pyrin) and inflammatory caspases, in the presence or absence of the inflammasome adaptor protein apoptosis-associated speck-like protein (ASC) [33]. The NLR protein-3 (NLRP3) inflammasome is currently the most well-characterized inflammasome. Diverse stimuli, including pathogen-associated molecular patterns and damage-associated molecular patterns (DAMPs), can activate the NLRP3 inflammasome, which consists of NLRP3, ASC, and pro-caspase-1. Once activated, the NLRP3 inflammasome can recruit pro-caspase-1. Procaspase-1 activation leads to its auto-cleavage into its p20 and p10 subunits, which then cause cleavage of interleukin-1β (IL-1β) and interleukin-18 (IL-18), and cleavage of gasdermin D, generating an N-terminal fragment that oligomerizes to form pores on the host cell membrane, leading to the lytic demise of the cell and release of cytokines [18]. In recent years, the NLRP3 inflammasome has been shown to contribute to the development of many cardiovascular diseases, including diabetic cardiomyopathy, myocardial infarction, and MI/R injury [48], as well as myocarditis [55]. Inflammasome activation serves as a crucial innate immune mechanism that protects the host from a wide variety of viral infections. Previous studies reported that the NLRP3 inflammasome participates in coxsackievirus B3-induced myocarditis, and modulation of inflammasome activation may be a promising therapeutic strategy for viral myocarditis [55]. However, the precise mechanisms of regulation of NLRP3 inflammasome in the development of viral myocarditis remain largely unknown.

The calpains are a well-conserved family of calcium-dependent cysteine proteases that are expressed ubiquitously in all cells [15]. Calpains operate by processing proteins via interacting with a limited number of motifs to alter their structure and function [25]. At least 16 calpains have been described, of which calpain-1 and calpain-2 are most well characterized. Calpain activity in vivo is tightly regulated by its natural endogenous inhibitor calpastatin [46]. In a previous study, we demonstrated that calpain inhibition prevented viral replication and myocardial injury in CVB3-induced myocarditis [3]; however, the specific roles of calpain subtypes in myocarditis and whether calpain acts in specific organelles remain to be explored. Activated calpain has been shown to release a pool of caspase-1 sequestered by the cytoskeleton to regulate NLRP3 activation [62]. As a result, we hypothesized that calpain-mediated regulation of the NLRP3 inflammasome may be involved in the pathogenesis of CVB3-induced myocarditis.

Therefore, in this study, we aimed to unveil the regulatory mechanism of calpain in the development of CVB3-induced myocarditis and explore the specific target of calpain, which may offer insights into novel cardioprotective therapeutic approaches for myocarditis treatment.

## Methods

### Virus and reagents

CVB3 used in this study was derived from the infectious cDNA copy of the cardiotropic Nancy strain and maintained at the Key Laboratory of Viral Heart Diseases, Zhongshan Hospital, Shanghai Medical College of Fudan University. Virus titer was routinely determined prior to infection by a 50% tissue culture infectious dose (TCID50) assay of Hela cell monolayer.

MCC950 (Catalog no.: HY-12815), Mito-TEMPO (Catalog no.: HY-112879), and PD151746 (Catalog no.: HY-19749) were purchased from MedChemExpress (America, New Jersey) and dissolved as per the manufacturer’s instructions. The concentrations of the drugs used in this study were as follows: MCC950, 10 mg/kg i.p. daily for 6 days; Mito-TEMPO, 0.7 mg/kg i.p. daily for 6 days; PD1517446, 20 μmol/L; Mito-TEMPO, 10 nM for cells.

The cell counting kit-8, enhanced ATP assay kit, and mitochondrial membrane potential assay kit with JC-1 were purchased from Biyotime (China). Cardiomyocytes isolation kit, DHE (Dihydroethidium) assay kit, Lipofectamine™ RNAiMAX, and MitoSOX™ red mitochondrial superoxide indicator were purchased from Invitrogen (America). Mitochondria isolation kit for tissue or cultured cells was purchased from Abcam (America). ELISA kit for mouse creatine kinase-MB (CK-MB), lactate dehydrogenase (LDH), cardiac troponin I (cTnI), IL-18, and IL-1β were purchased from MULTI SCIENCES (China).

### Animals

The calpastatin transgenic mouse strain (Tg-CAST; C57BL/6 background) was introduced from the laboratory of Sidong Xiong (Soochow University, China) and was crossed with Balb/c background mice for at least eight generations to obtain transgenic mice with Balb/c background. All littermates were genotyped following a previous protocol [4]. Male mice were used. To induce viral myocarditis, mice aged 4–5 weeks were intraperitoneally injected with CVB3 (105 TCID50, 300 μL) as previously described [28], while mice in the sham group were injected with the same amount of phosphate-buffered saline (PBS). In mice co-treated with inhibitors, inhibitors were injected intraperitoneally, starting at 2 days before CVB3 infection. Mice were divided into the following three sets of groups:

1. Wild-type (WT) mice intraperitoneally injected with PBS (Sham group), or CVB3 (CVB3 group), or MCC950 followed by CVB3 (CVB3 + MCC950 group).
2. WT mice or Tg-CAST mice intraperitoneally injected with PBS (WT + sham group and Tg-CAST + sham group, respectively), or with CVB3 (WT + CVB3 group and Tg-CAST + CVB3 group, respectively).
3. WT mice intraperitoneally injected with PBS (Sham group) or CVB3 (CVB3 group), or mito-TEMPO followed by CVB3 (CVB3 + mito-TEMPO group).

All mice were bred in the Department of Laboratory Animal Science, Fudan University in a standard specific pathogen-free (SPF) environment, and all animal experiments were performed in accordance with the local institutional guidelines and regulations of the Ethical Committee of Fudan University and conformed to the Directive 2010/63/EU of the European Parliament. Mice were euthanized by pentobarbital (150 mg/kg) overdose by intraperitoneal injection.

### Cell isolation and culture

Neonatal rat cardiomyocytes (NRCMs) were isolated from 1- to 3-day-old Sprague–Dawley rats using a cardiomyocyte isolation kit (Thermo Fisher Scientific, America) according to the manufacturer’s instructions. Briefly, hearts were removed from the thorax after euthanizing mice by decapitation, and the ventricles were finely minced and digested in the mixed enzyme solution from the kit for 30–35 min. To remove cardiac fibroblasts, dispersed cells were pre-plated for 1.5 h, and the supernatant containing cardiomyocytes was separated and cells were cultured in Dulbecco’s modified Eagle’s medium (DMEM) supplemented with 10% fetal bovine serum (LONSERA A511-001, ShuangRu Biotech.), 1% penicillin/streptomycin, 10 μM cytosine 1-β-d-arabinofuranoside (Ara C) at 37 °C in a 5% CO2 incubator. The NRCMs were seeded at a density of 1.2 × 10^5^/cm^2^, except for immunofluorescence staining, in which the cell density was 0.8 × 10^5^/cm^2^. After 24 h, the media were replaced with culture media (M199 media supplemented with 1% ITS, 1% penicillin/streptomycin, and 10 μM Ara C). The human cardiomyocyte cell line AC16 was cultured in DMEM supplemented with 10% FBS and 1% penicillin/streptomycin. For CVB3 infection, NRCMs or AC16 cells were incubated with CVB3 in serum-free media for 2 h at 37 °C, following which they were washed with PBS and cultured in culture media.

### Western blot analysis

After different treatments, the cells were incubated with RIPA lysis buffer containing a protease inhibitor cocktail on ice for 10 min. Cell lysates were then harvested by scraping, followed by brief sonication and centrifugation at 12,000 × g for 10 min at 4 °C. The protein concentration was determined via the BCA assay. Equal amounts of protein were separated by SDS/PAGE and the bands were electroblotted onto PVDF membranes. The membranes were blocked with 5% non-fat milk dissolved in TBST and then incubated with appropriate primary antibodies overnight at 4 °C. The following primary antibodies were used: anti-NLRP3 (1:1000, Abcam), anti-Gasdermin D-N-terminal (1:1000, Invitrogen), anti-caspase-1 (1:1000, Invitrogen), anti-cleaved caspase-1 (1:1000, Invitrogen), anti-GAPDH (1:10000, Abcam), α-Fodrin (1:1000, Cell Signaling Technology), anti-ATP5A1 (1:1000, Invitrogen), Calpastatin (1:500, ABclonal), and Flag (1:1000, Abcam). The membranes were then incubated with the following horseradish peroxidase-conjugated secondary antibodies: HRP-labeled goat anti-rabbit IgG and HRP-labeled goat anti-mouse IgG purchased from Proteintech (1:5000, America). The immunofluorescent bands were then visualized and quantified using an ECL chemiluminescence kit (Absin, Shanghai, China) and a chemiluminescence–western blotting detection system (Tanon, Shanghai, China).

### Immunofluorescence or immunohistochemistry staining

Paraffin-embedded heart tissues were cut into 3-µm-thick sections, deparaffinized, and rehydrated using xylol and a graded alcohol series prior to staining, after which antigen retrieval was performed using antigen retrieval solution (Beyotime, China) for 20 min at 98 °C. The slides were then blocked with 1% BSA in PBS at room temperature for 30 min and incubated with a primary antibody at 4 °C overnight. For immunofluorescence staining, fluorescence-labeled secondary antibodies were added and incubated at room temperature for 1 h. The cell nucleus was visualized using DAPI staining. For immunohistochemistry staining, endogenous tissue peroxidase activity was quenched with 3% H_2_O_2_ for 20 min. The slides were then treated with the Vectastain Elite ABC Kit (Avidin/Biotin/Horseradish Peroxidase-System (Vector Laboratories) after primary antibody incubation. The peroxidase reaction was visualized using 3,3′-diaminobenzidine tetrahydrochloride (DAB) and slides were counterstained with hematoxylin. For cell staining, cardiomyocytes were plated in 35-mm glass dishes and subjected to the designed treatments. The cells were then washed with PBS and fixed with 4% paraformaldehyde for 20 min at room temperature, following which they were washed with PBS three times, incubated with 0.2% Triton X-100 for permeabilization for 15 min, and then blocked in 1% BSA for 1 h. The cells were subsequently incubated with specific primary antibodies overnight at 4 °C. The next day, the cells were washed with PBS, incubated with fluorescence-conjugated secondary antibodies (Beyotime, China), and subjected to DAPI (5 ug/mL, share-bio, China) staining. Images were captured using a fluorescence microscope. The primary antibodies used with dilution factors are as follows: anti-NLRP3 (1:200, Abcam), anti-ASC (1:200, Abcam), anti-cleaved caspase-1 (1:200, Invitrogen), anti-calpain-1 (1:200, Invitrogen), anti-COX IV (1:200, Invitrogen), anti-CD4 (1:200, Abcam), and anti-F4/80 (1:200, Abcam).

### Transmission electron microscopy

Ventricular tissues were fixed in 2.5% glutaraldehyde/0.05 mol/L cacodylate solution, postfixed with 1% osmium tetroxide, and embedded in EmBed812. Ultrathin sections (70 nm) were poststained with uranyl acetate and lead citrate and examined using the Talos F200X FEG transmission electron microscope (FEI, Hillsboro, OR) at 80 kV. Digital electron micrographs were recorded using the TIA software (FEI).

### Hematoxylin–eosin (HE) and Masson’s trichrome staining

HE and Masson’s trichrome staining were performed as previously described [32]. Mice hearts were embedded in paraffin and then cut into 5-μm-thick serial sections. The sections were dyed with HE and Masson’s trichrome using standard protocols.

### TUNEL staining

Terminal deoxynucleotidyl transferase-mediated dexoxyuridine triphosphate nick-end labeling (TUNEL) staining was performed using the In Situ Cell Death Detection Kit (Roche, Switzerland) following the manufacturer’s instructions. Apoptotic nuclei were labeled with green fluorescein staining and total cardiomyocyte nuclei were stained with DAPI. The pictures of heart tissues were obtained using immunofluorescence microscopy. The rate of apoptosis was calculated as the ratio of TUNEL-positive nuclei to DAPI-stained nuclei.

### Echocardiography

Echocardiography was performed under anesthesia using the Vevo770 imaging system (VisualSonics Inc., Toronto, ON, Canada) with a 30-MHz high-frequency scan head, and the ejection fraction (EF) and fractional shortening (FS) were obtained.

### Detection of reactive oxygen species (ROS)

Hearts were perfused with ice-cold PBS and removed quickly, following which the hearts were embedded in OCT and snap-frozen in liquid nitrogen. 5-µm-Thick sections were then stained with dihydroethidium (DHE, Invitrogen) for 15 min followed by DAPI staining (1 µg/ml, Sigma). Pictures were captured using a fluorescence microscope. The percentage of DHE-positive nuclei was calculated as the indication of ROS levels.

### Small interfere RNA transfection

Downregulation of rat ATP5A1 in NRCMs was achieved using the following siRNA from RIBOBIO: GATCATCTATGACGACTTA. NRCM suspension was transfected with siRNA-ATP5A1 (siATP5A1) or siRNA-Scramble (siCT) when plated following Lipofectamine™ RNAiMAX as the reverse transfection protocol.

### Adenoviral constructions and transfection

To overexpress calpain-1 in NRCMs, the full-length cDNA sequence of rat calpain-1 catalytic subunit (CAPN1) was inserted into the pADV-mCMV-MCS-3xFLAG adenoviral vector from OBiO Technology. NRCMs were infected with adenovirus containing CAPN1 (Ad-CAPN1) and the control adenovirus (Ad-CT) in media without penicillin/streptomycin or FBS for 6 h, and the media were then changed to culture media for 48 h before the next operation.

### Measurement of oxygen consumption rate

XFe96 extracellular flux analyzer (Seahorse Bioscience) was used to measure the oxygen consumption rate (OCR) of NRCMs after indicated treatments as described previously [47]. Briefly, NRCMs were plated on a 1% gelatin-coated Seahorse 96-well plate at a density of 1.2 × 105/cm^2^. After the NRCMs were treated, metabolic profiles of OCR were detected by adding 1 µM oligomycin (Oligo), 1 µM FCCP, and a mix of 1 µM rotenone and 1 µM antimycin A (RAA). Oxidative phosphorylation indexes were calculated as follows:

Basal respiration = (average OCR pre-Oligo)—(average OCR post- RAA).

ATP-linked respiration = (average OCR pre-Oligo)—(average OCR post-Oligo).

Maximal respiration = (average OCR post-FCCP)—(average OCR post-RAA).

### Co-immunoprecipitation

NRCMs were lysed using NP40 lysis buffer supplemented with protease inhibitor. The cell lysates were mixed with anti-IgG (cell signaling technology), anti-calpain-1 (Invitrogen), anti-Flag (Abcam), and anti-ATP5A1 (Abcam) antibody that was preincubated with Protein A/G Magnetic Beads (MedChemExpress) overnight at 4 °C. Coprecipitates with primary antibody were separated by SDS-PAGE followed by mass spectrometry or incubation with non-heavy chain IgG secondary antibody.

### Statistical analysis

Data are presented as the means ± SEM. Statistical analysis between groups was performed using an unpaired Student’s t test. Differences among multiple groups were tested using one-way ANOVA or two-way ANOVA, followed by Bonferroni’s post hoc test using GraphPad Prism (GraphPad Prism 8 Software Inc, San Diego, CA). Differences were considered significant at *P* < 0.05, *P* < 0.01, *P* < 0.001, and *P* < 0.0001.

## Results

### MCC950 inhibited CVB3-induced pyroptosis and inflammation and preserved cardiac function

**Fig. 1:**
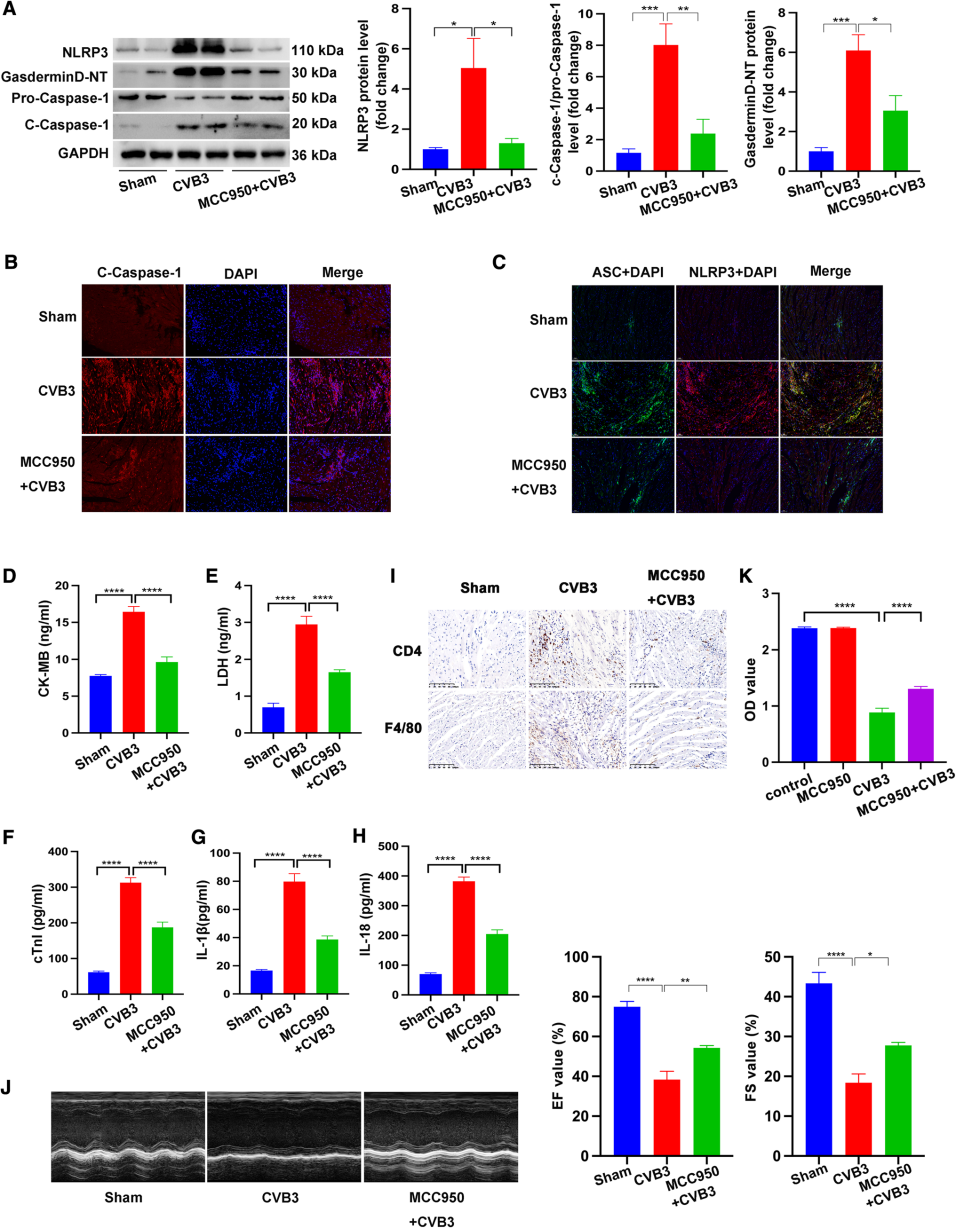
Mouse models of myocarditis were established by intraperitoneally injecting mice with CVB3. To inhibit NLRP3 inflammasome activation in CVB3-infected mice, MCC950 was administrated intraperitoneally at a dose of 10 mg/kg daily starting at the 2 days before CVB3 treatment. Assays were carried out at 7 days after CVB3 infection. **A** Representative image of western blotting results and the quantitative analysis of NLRP3, Gasdermin D-NT, pro-caspase 1, c-caspase 1 in heart tissues. **B**, **C** Representative images of immunofluorescence staining showing the expression of c-caspase-1, NLRP3, and ASC in heart sections. **D**, **E**, **F**, **G**, **H** Levels of serum CK-MB, LDH, cTnI, IL-18, and IL-1β in mice subject to the indicated treatments. **I** Representative images of immunohistochemistry staining showing the cardiac infiltration of CD4-positive T lymphocytes and F4/80-positive macrophages. **J** Echocardiography images and quantitative statistics of ejection fraction (EF) and fractional shortening (FS). *N* = 6 mice in each group. **K** NRCMs were treated with MCC950 or CVB3 infection or both MCC950 and CVB3. After 48 h, CCK8 was used to assess cell viability in each group. *N* = 16 replicates from three independent experiments

Although NLRP3 inflammasome activation and pyroptosis have been reported in the pathology of CVB3-induced myocarditis, direct inhibition of NLRP3 inflammasome to investigate the role of NLRP3 in myocarditis has not been conducted. Administration of MCC950, a selective inhibitor of the NLRP3 inflammasome, before CVB3 infection, led to decreased protein levels of NLRP3, N-terminal domain of Gasdermin D (Gasdermin D-NT), and cleaved caspase-1 (c-caspase-1) (Fig. 1A). Immunofluorescence density of c-caspase-1 was decreased in the heart sections from mice treated with MCC950 and CVB3 compared to that from CVB3 infection alone, consistent with the result from western blot (Fig. 1B). Importantly, co-localization of NLRP3 and ASC was inhibited significantly with MCC950 administration, suggesting the reduced platform formation of NLRP3 inflammasome (Fig. 1C). Pyroptosis status was assessed using ELISA assay, and showed that levels of serum CK-MB, LDH, cTnI, IL-18, and IL-18β were increased after CVB3 stimulation, but these effects were reversed by MCC950 treatment (Fig. 1D, E, F, G, H). Furthermore, the infiltration of CD4-positive T lymphocytes and F4/80-positive macrophages induced by CVB3 infection was less obvious in the CVB3 + MCC950 group (Fig. 1I). Furthermore, the CVB3 + MCC950 group showed significantly improved cardiac function by decreasing the ejection factor (EF) and fractional shortening (FS) compared with the CVB3 group (Fig. 1J). Moreover, cardiomyocyte viability was higher in the CVB3 + MCC950 than in the CVB3 group, suggesting that MCC950-mediated inhibition of NLRP3 inflammasome activation reduced CVB3-induced death (Fig. 1K).

### Calpastatin overexpression inhibited CVB3-induced apoptosis, fibrosis, and inflammation, and improved cardiac dysfunction

**Fig. 2:**
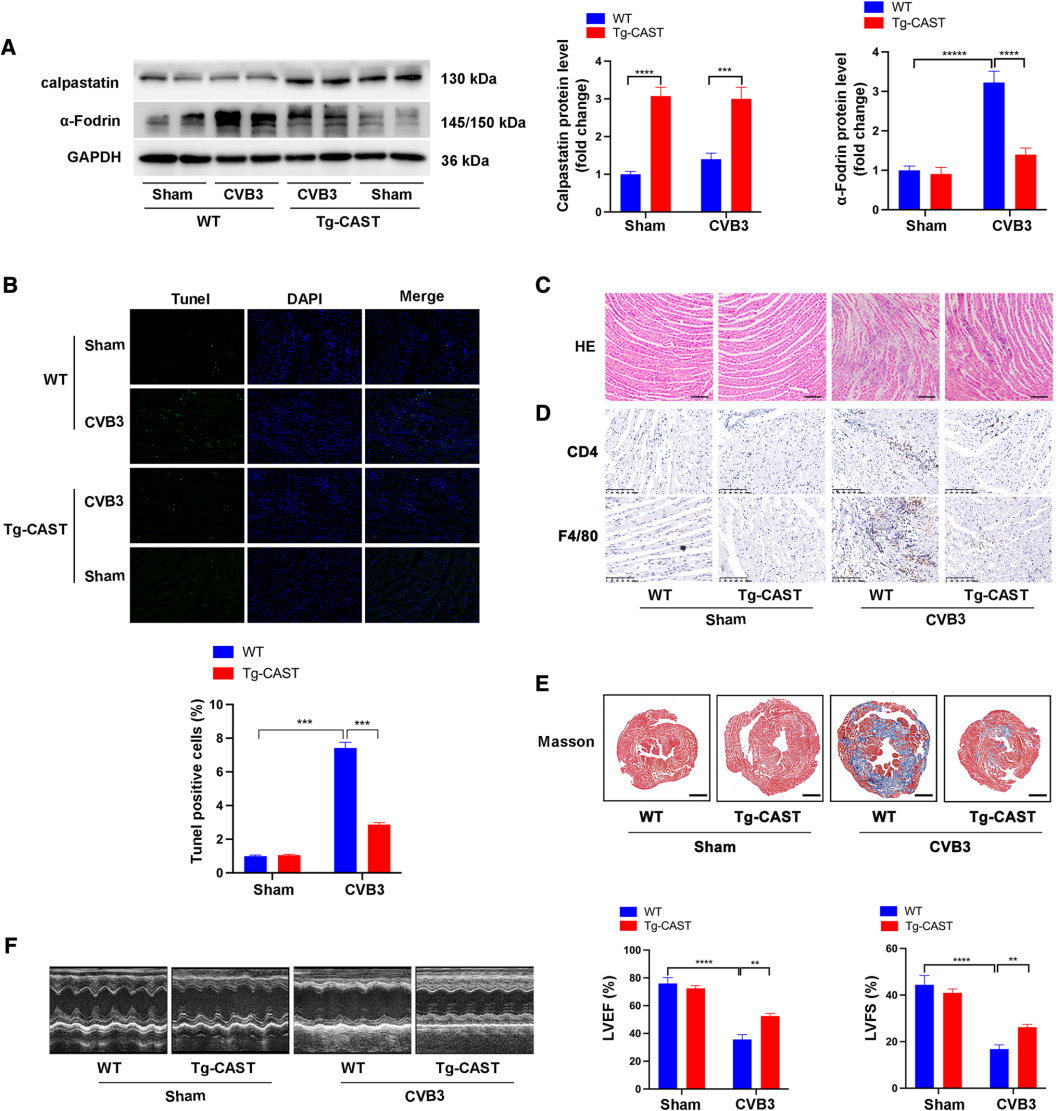
CVB3-induced myocarditis models were constructed in calpastatin overexpression mice (Tg-CAST) and their wild-type littermates (WT). **A** Representative image of western blotting results and the quantitative analysis of calpastatin and α-Fodrin after CVB3 infection for 7 days. **B** Representative images and quantification of TUNEL-positive cells in heart section after CVB3 infection for 7 days. Data were collected from 18 fields from six mice in each group. **C** HE staining of heart sections showing local changes in different mice after CVB3 infection for 7 days; bar = 100 µm. **D** Representative images of immunohistochemistry staining showing cardiac infiltration of CD4 positive T lymphocytes and F4/80-positive macrophages after CVB3 infection for 7 days; bar = 100 µm. **E** Masson’s trichrome staining of heart sections showing fibrosis after CVB3 infection for 7 days.; bar = 500 mm. **F** Echocardiography images and quantitative statistics of EF and FS. *N* = 6 mice in each group

To further uncover the mechanism underlying the effects of calpain in CVB3-induced myocarditis, we induced myocarditis in Tg-CAST mice overexpressing the calpain inhibitor calpastatin and their WT littermates by CVB3 injection. High expression of calpastatin in heart tissue from Tg-CAST mice was validated first (Fig. 2A). As α-Fodrin at 145/150 kDa is the degradation product of activated calpain, the levels of α-Fodrin at 145/150 kDa were detected to confirm the activation of calpain. As shown in Fig. 2A, on day 7 post-CVB3 injection, α-Fodrin at 145/150 kDa was substantially increased in the WT + CVB3 group, while it was decreased in the Tg-CAST + CVB3 group, suggesting the inhibitory effect of calpastatin overexpression on calpain activation. TUNEL staining showed an increased number of TUNEL-positive cells in the WT + CVB3 group than in the Tg-CAST + CVB3 group (Fig. 2B). Results from HE staining indicated that calpastatin overexpression inhibited CVB3-induced inflammatory responses presented by less infiltrating inflammatory cells and necrosis foci in the Tg-CAST + CVB3 group than in the WT + CVB3 group (Fig. 2C). The infiltration of CD4-positive T lymphocytes and F4/80-positive macrophages after CVB3 infection was also decreased in heart tissues from Tg-CAST mice than in those from WT mice (Fig. 2D). Long-term CVB3 infection leads to cardiac fibrosis and cardiac dysfunction. Masson staining of heart tissue sections (Fig. 2E) revealed that cardiac fibrosis increased remarkably 1 month post-CVB3 infection in WT mice but not in Tg-CAST mice. Further assessment of cardiac function via echocardiography reinforced this finding. CVB3 infection led to obvious cardiac dysfunction in WT mice, presented as decreased left ventricle EF and FS, whereas calpastatin overexpression in Tg-CAST mice remarkably improved cardiac function (Fig. 2F). Therefore, calpain inhibition in vivo not only protected mice hearts from CVB3 infection-induced cell apoptosis and inflammatory responses in the acute phase but also protected mice hearts from cardiac fibrosis and cardiac dysfunction in the long term.

### Calpastatin overexpression inhibited NLRP3 inflammasome and pyroptosis after CVB3 infection

**Fig. 3:**
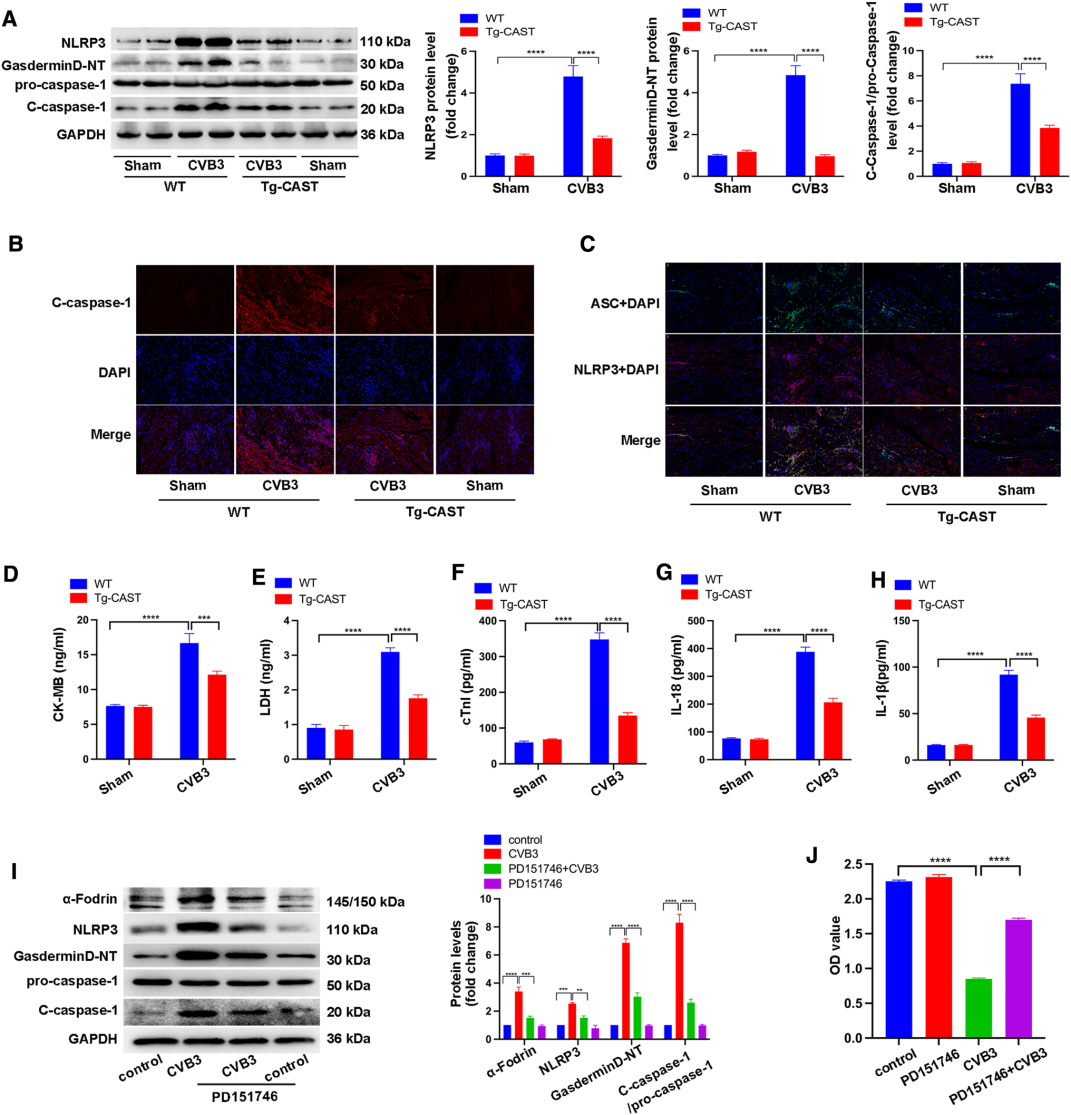
NLRP3 inflammasome activation and pyroptosis in Tg-CAST and WT mice with or without CVB3 infection. **A** Representative image of western blotting results and the quantitative analysis of NLRP3, gasdermin D-NT, pro-caspase 1, and c-caspase 1 in heart tissues. **B**, **C** Representative images of immunofluorescence staining showing the expression of c-caspase-1, NLRP3, and ASC in each group. **D**, **E**, **F**, **G**, **H** Serum levels of CK-MB, LDH, cTnI, IL-18, and IL-1β. *N* = 6 mice in each group. NRCMs were treated with PD151746 or CVB3 or both PD151746 and CVB3 for 48 h. **I** Representative image of western blotting results and the quantitative analysis of α-Fodrin, NLRP3, gasderminD-NT, pro-caspase-1, and c-caspase-1. *N* = 3 independent experiments. **G** CCK8 showing the viability of NRCMs after the indicated treatments. *N* = 24 from three independent experiments

Next, we determined whether calpain played a role in the activation of the NLRP3 inflammasome. Tg-CAST mice showed decreased levels of NLRP3, gasderminD-NT, and c-caspase-1 significantly, compared to WT mice after CVB3 infection (Fig. 3A). Immunofluorescence staining also showed that calpastatin overexpression in the Tg-CAST + CVB3 group inhibited the expression of c-caspase-1, ASC, NLRP3, and the co-localization of ASC and NLRP3 (Fig. 3B, C). Moreover, the Tg-CAST + CVB3 group showed lower levels of serum CK-MB, LDH, cTnI, IL-18, and IL-1β, indicative of less pyroptosis (Fig. 3D, E, F, G, H). To validate our findings in vivo and determine if calpain-1 was the dominant calpain in CVB3-induced myocarditis, we treated neonatal rat cardiomyocytes with the calpain-1 selective inhibitor PD151746 after CVB3 infection. PD151746 treatment decreased the levels of α-Fodrin at 145/150 kDa, confirming that the activation of calpains was strikingly inhibited by PD151746 (Fig. 3I). Furthermore, PD151746 treatment decreased the levels of NLRP3, Gasdermin D-NT, and c-caspase-1 that were elevated in cardiomyocytes after CVB3 infection (Fig. 3I). Additionally, PD151746 protected cardiomyocytes from CVB3-induced cell death (Fig. 3J). These findings confirmed that calpain-1 is the main calpain in CVB3-induced myocarditis. Overall, the results of in vivo and in vitro experiments suggest that inhibition of the activation of calpains in the setting of CVB3 infection could block the activation of the NLRP3 inflammasome and suppress pyroptosis.

### Mitochondrial-targeted antioxidant (mito-TEMPO) prevented inflammation, inhibited the NLRP3 inflammasome, and improved cardiac function

**Fig. 4:**
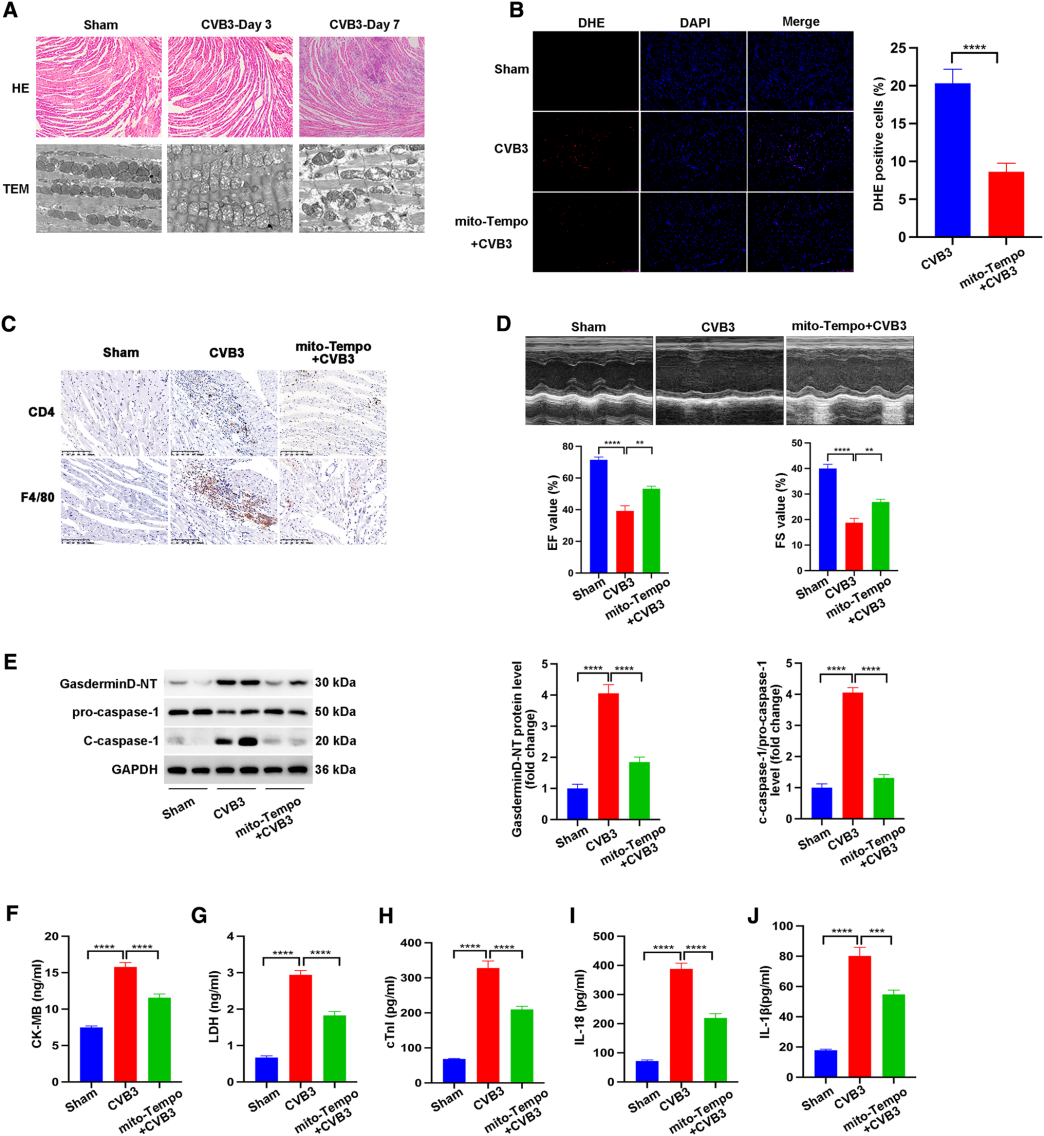
To determine whether mitochondrial ROS participated in CVB3-induced myocarditis, mito-TEMPO at a dose of 0.7 mg/kg daily was administrated intraperitoneally starting at the 2 days before CVB3 treatment. Assays were conducted after CVB3 infection. **A** Representative images of HE staining and transmission electron microscopy showing changes in the histology and mitochondria of the heart on 3rd day or 7th day after CVB3 treatment. **B** DHE staining and quantification of positive DHE in nuclei. *N* = 4 mice from each group. **C** Representative images of immunohistochemistry staining showing cardiac infiltration of CD4-positive T lymphocytes and F4/80-positive macrophages. **D** Echocardiography images and quantitative analysis of EF and FS. **E** Representative image of western blotting results and quantitative analysis of gasdermin D-NT, pro-caspase-1, and c-caspase-1. **F**, **G**, **H**, **I**, **J** Serum levels of CK-MB, LDH, cTnI, IL-18, and IL-1β in mice subjected to different treatments. *N* = 6 mice in each group

The heart is the most energy-consuming organ, and mitochondria are essential for the maintenance and development of the myocardium [39]. Mitochondria integrate diverse signals and relay this information to the NLRP3 inflammasome, leading to downstream inflammatory responses [18]. Therefore, we further investigated the effects of CVB3 on mitochondria function during the development of viral myocarditis. HE staining revealed that on day 3 post-CVB3 infection, infiltrating inflammatory cells and necrosis foci could hardly be found, but on day 7 post-CVB3 infection, the myocardium presented severe cardiac inflammation and injury (Fig. 4A). However, transmission electron microscopy revealed that the alteration of mitochondrial morphology started on day 3 post-CVB3 infection. The mitochondria were swollen showing the irregular arrangement and contained irregularly arranged cristae, abnormal cristae, and an increased number of vacuoles (Fig. 4A). Therefore, the appearance of abnormal mitochondria, indicative of mitochondrial dysfunction, ahead of the inflammatory response may lead to inflammation in CVB3-induced myocarditis. Mitochondrial reactive oxygen species (ROS) production occurs after mitochondrial dysfunction, and ROS may serve as signaling molecules triggering pro-inflammatory responses in cardiomyocytes [63]. mito-TEMPO is a nitroxide conjugated with a triphenylphosphonium moiety and a superoxide dismutase-mimetic and acts like a mitochondrial superoxide scavenger. mito-TEMPO is used as a mitochondria-targeted antioxidant in cardiovascular diseases [6]. Intraperitoneal treatment of mice with mito-TEMPO before CVB3 infection decreased CVB3-induced mitochondrial ROS levels (Fig. 4B). Infiltration of CD4-positive T lymphocytes and F4/80-positive macrophages induced by CVB3 infection was also blocked by mito-TEMPO (Fig. 4C). Importantly, mito-TEMPO improved cardiac function, indicated by enhanced EF value and FS value (Fig. 4D). We then investigated if mitochondrial ROS was a causing factor of NLRP3 inflammasome activation. mito-TEMPO treatment decreased not only the CVB3-induced increase in the levels of gasdermin D-NT and c-caspase-1 (Fig. 4E) but also the increased levels of serum CK-MB, LDH, IL-18, and IL-1β (Fig. 4F, G, H, I, J). In conclusion, these results suggest an association between mitochondrial ROS and NLRP3 inflammasome activation in CVB3-induced myocarditis.

### Calpain-1 regulated mitochondrial function and mito-TEMPO reversed the calpain-1-mediated NLRP3 inflammasome activation

**Fig. 5:**
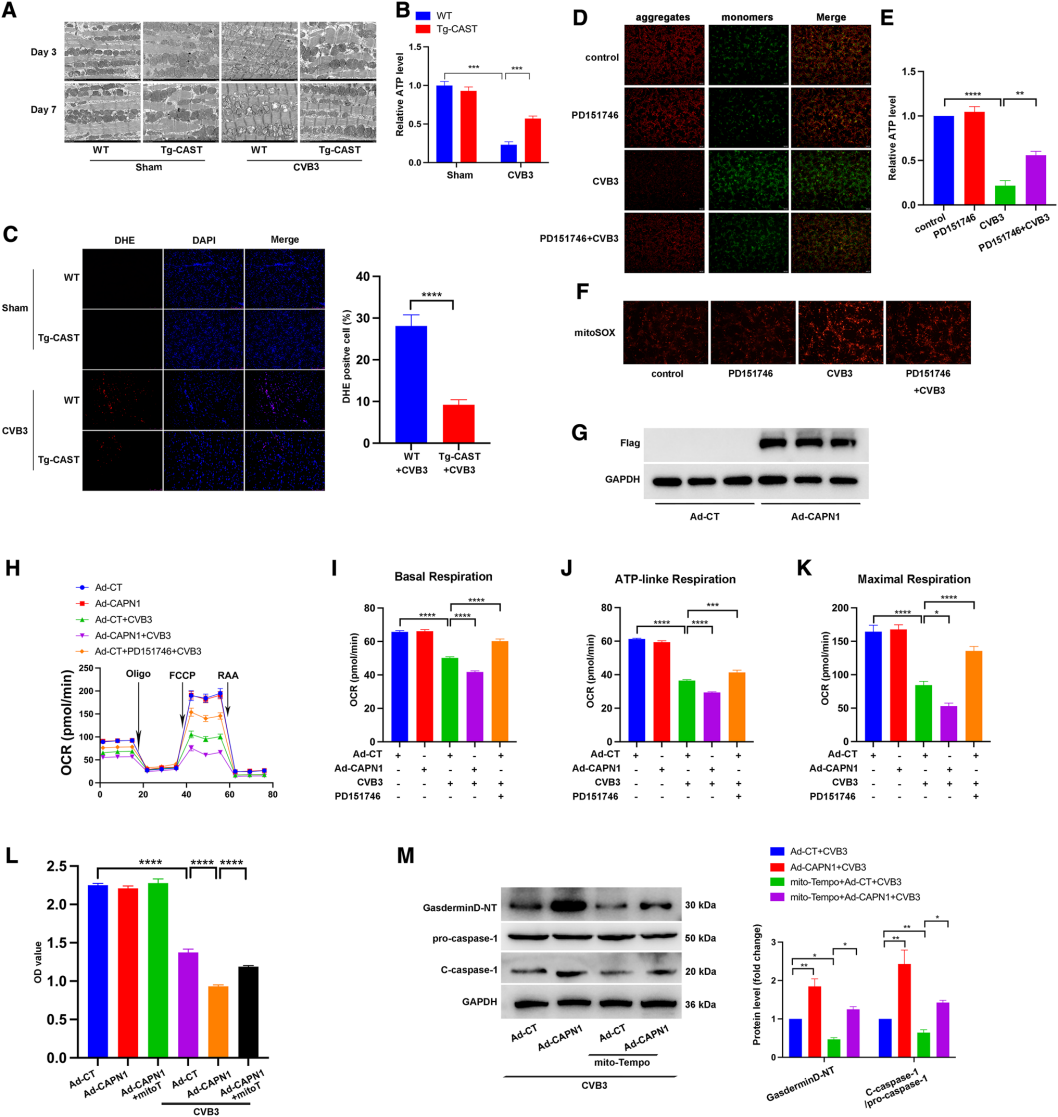
**A** Transmission electron microscopy images showing the structure of cardiac mitochondria subjected to different treatments. **B** Relative ATP levels in the heart of WT or Tg-CAST mice with or without CVB3 infection for 7 days. *N* = 6 mice in each group. **C** Representative images of DHE staining. After oxidization, DHE intercalates into DNA and stains the nuclei red. The percentages of DHE-positive cells were quantified. *N* = 4 mice. **D** Representative images of JC-1 staining of NRCMs showing the mitochondrial membrane potential in each group. *N* = 3 independent experiments. **E** Relative ATP levels in NRCMs after treatment with CVB3 or PD151746. *N* = 3 independent experiments. **F** Representative images of mitochondrial ROS in NRCMs stained by mitoSOX indicator. *N* = 3 independent experiments. **G** NRCMs were infected with CAPN1-expressing adenovirus (Ad-CAPN1) or control adenovirus (Ad-CT) at an MOI (multiplicity of infection) of 50 for 48 h, and the Flag-tag was then detected by western blotting to show the effective infection of Ad-CAPN1. *N* = 3 independent experiments. **H** NRCMs were infected with Ad-CT or Ad-CAPN1 for 48 h with or without CVB3 treatment. PD151746 was added to some cells to inhibit the activation of calpain-1. After treatment, oxygen consumption rate (OCR) was detected using the Seahorse platform. **I** Basal respiration; **J** ATP-linked respiration; **K** maximal respiration. *N* = 17 replicates in each group. **L** NRCMs were infected with Ad-CT or Ad-CAPN1 for 48 h with or without CVB3 treatment. Mito-TEMPO was added to NRCMs treated with Ad-CAPN1 and CVB3. CCK8 shows cell viability. *N* = 16 replicates from three independent experiments. **M** Representative image of western blotting bands and the quantitative analysis of gasdermin D-NT, pro-caspase-1, and c-caspase-1 under different treatments. *N* = 3 independent experiments

As our results suggested an association between mitochondria dysfunction and inflammatory response in CVB3-induced myocarditis, we examined the role of calpain in these processes. Transmission electron microscopy revealed that day 3 onwards, the Tg-CAST + CVB3 group overexpressing calpastatin showed alleviated mitochondria structural damage, and well-organized mitochondria with more regularly arranged cristae and fewer vacuoles compared to the WT + CVB3 group (Fig. 5A). Moreover, as shown, CVB3 infection significantly decreased cardiac ATP production, while calpastatin overexpression reversed the impaired ATP production, although not significantly (Fig. 5B). Besides, cardiac ROS staining was less obvious in the Tg-CAST + CVB3 than in the WT + CVB3 group (Fig. 5C). The above results suggested a better mitochondrial function when calpain activity was inhibited in vivo. Furthermore, we also assessed mitochondrial function after CVB3 infection under the treatment of calpain-1 inhibitor, PD151746. Mitochondrial membrane potential (Δψm) measurements by JC-1 staining showed that CVB3 infection led to decreased Δψm indicated by a higher ratio of green/red fluorescence and the sole treatment of PD151746 did not affect Δψm, whereas PD151746 treatment prevented Δψm loss under CVB3 infection (Fig. 5D). In addition, CVB3 infection decreased ATP production in cardiomyocytes, but PD151746 treatment reserved ATP production to a certain degree under CVB3 infection (Fig. 5E). Next, we used MitoSOX Red probes to assay mitochondrial oxidative stress. As shown in Fig. 5F, CVB3 increased mitochondrial ROS production in cardiomyocytes, which could be inhibited by PD151746. To detect the effects of Calapin-1 in cardiomyocytes more directly, we infected primary cardiomyocytes with adenovirus overexpressing calpain-1 conjugated with Flag (Ad-CAPN1). Ad-CAPN1 increased Flag expression dramatically, indicating high infection efficiency (Fig. 5G). Cardiomyocytes were treated with Ad-CAPN1 or control adenovirus (Ad-CT) for 48 h and were then infected with CVB3, or Ad-CT-infected cardiomyocytes were treated with CVB3 and PD151746, following which the oxygen consumption rate (OCR) was measured using the XFe96 extracellular flux analyzer. Basal respiration, ATP-linked respiration, and the maximal respiration of mitochondria were inhibited under CVB3 treatment, while calpain-1 overexpression with Ad-CAPN1 infection deteriorated these effects further. In contrast, PD151746 alleviated mitochondrial respiration impaired by CVB3 (Fig. 5H, I, J, K). Moreover, calpain-1 overexpression aggravated CVB3-induced cell death, whereas mito-TEMPO preserved cell viability (Fig. 5L). Similarly, the protein levels of gasdermin D-NT and c-caspase-1 enhanced by calpain-1 overexpression were decreased after mito-TEMPO incubation under CVB3 treatment (Fig. 5M). In conclusion, calpain-1 regulated mitochondrial function under CVB3 treatment through mitochondrial ROS-mediated activation of the NLRP3 inflammasome.

### Calpain-1 accumulated in mitochondria after CVB3 stimulation

**Fig. 6:**
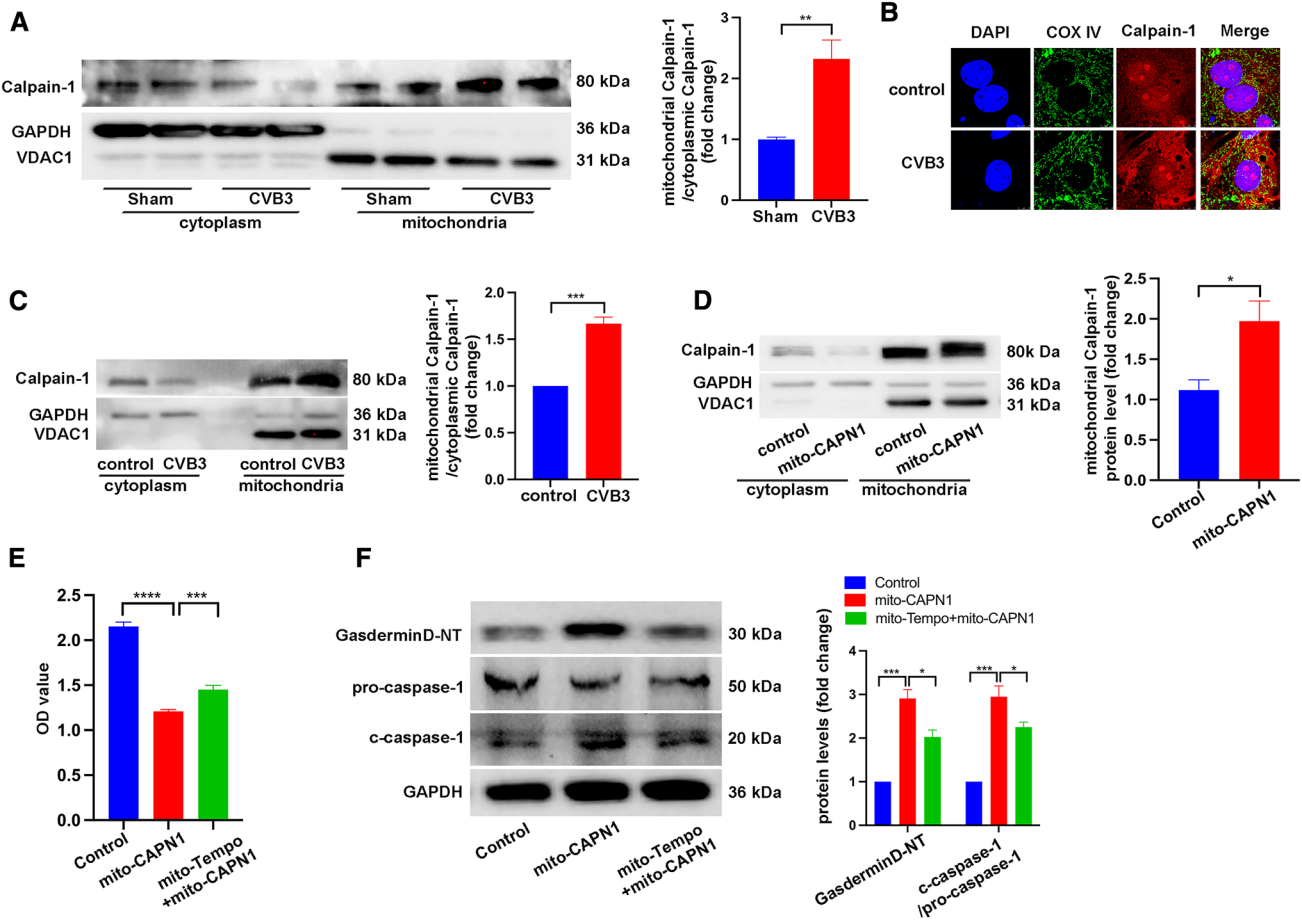
**A** Mitochondria was isolated from cytoplasm in CVB3-infected hearts or control hearts, and then, protein levels of calpain-1 were detected in each fraction. *N* = 6 mice in each group. **B** Immunofluorescence staining showing the co-localization of calpain-1 and mitochondria in NRCMs infected with CVB3. **C** calpain-1 levels in mitochondria isolated from CVB3-infected NRCMs. *N* = 3 independent experiments. **D** Representative image of western blotting results and the quantitative analysis of calpain-1 in cytoplasm or mitochondria in AC16 cells transfected with a plasmid expressing mitochondria-targeted calpain-1 (mito-CAPN1) or control plasmid. *N* = 3 independent experiments. **E** Viability of AC16 cells transfected with mito-CAPN1 and incubated with mito-TEMPO as determined by the CCK8 assay. *N* = 24 replicates from three independent experiments. **F** Representative image of western blotting results and the quantitative analysis of gasdermin D-NT, pro-caspase-1, and c-caspase-1. *N* = 3 independent experiments

We demonstrated that calpain inhibition improved mitochondrial dysfunction both in vivo and in vitro, which mediated its regulatory effects on NLRP3 inflammasome activation under CVB3 infection; however, the specific target of calpain-1 in cardiomyocytes remains unknown. Previous studies have described the mitochondrial localization of calpain members [16]; therefore, we assessed the cytosolic and mitochondrial localization of calpain-1 in cardiomyocytes under CVB3 infection. Cytosolic and mitochondrial fractions were separated in heart tissue, and then, the protein level of calpain-1 was detected. The purity of mitochondria was confirmed by the absence of GAPDH and the enrichment of VDAC1, and the ratio of mitochondrial calpain-1 to cytosolic calpain-1 was significantly higher in the CVB3-infected hearts (Fig. 6A). Consistent with the in vivo findings, immunofluorescence staining showed that CVB3 infection promoted the co-localization of calpain-1 and mitochondria in cultured cardiomyocytes (Fig. 6B) and that the protein levels of calpain-1 after CVB3 infection were higher in the mitochondria than in the cytosol (Fig. 6C). Thus, the redistribution of intracellular calpain-1 may contribute to its effects under CVB3 infection. Next, AC16 cells were transfected with pCMV/myc/mito-CAPN1 containing mitochondrial targeting signal or pCMV/myc/mito as a control. Calpain-1 overexpression was restricted in mitochondria (Fig. 6D). The overexpression of calpain-1 in mitochondria led to decreased AC16 cell viability, which could be blocked by mito-TEMPO (Fig. 6E). Furthermore, protein levels of gasdermin D-NT and c-caspase-1 were increased by mitochondrial calpain-1 overexpression but inhibited by mito-TEMPO (Fig. 6F). Taken together, our data indicated that the cytosolic-to-mitochondrial translocation of calpain-1 after CVB3 stimulation was responsible for its regulation on cell viability and NLRP3 inflammasome activation.

### ATP5A1 is the cleaving target of calpain-1

**Fig. 7:**
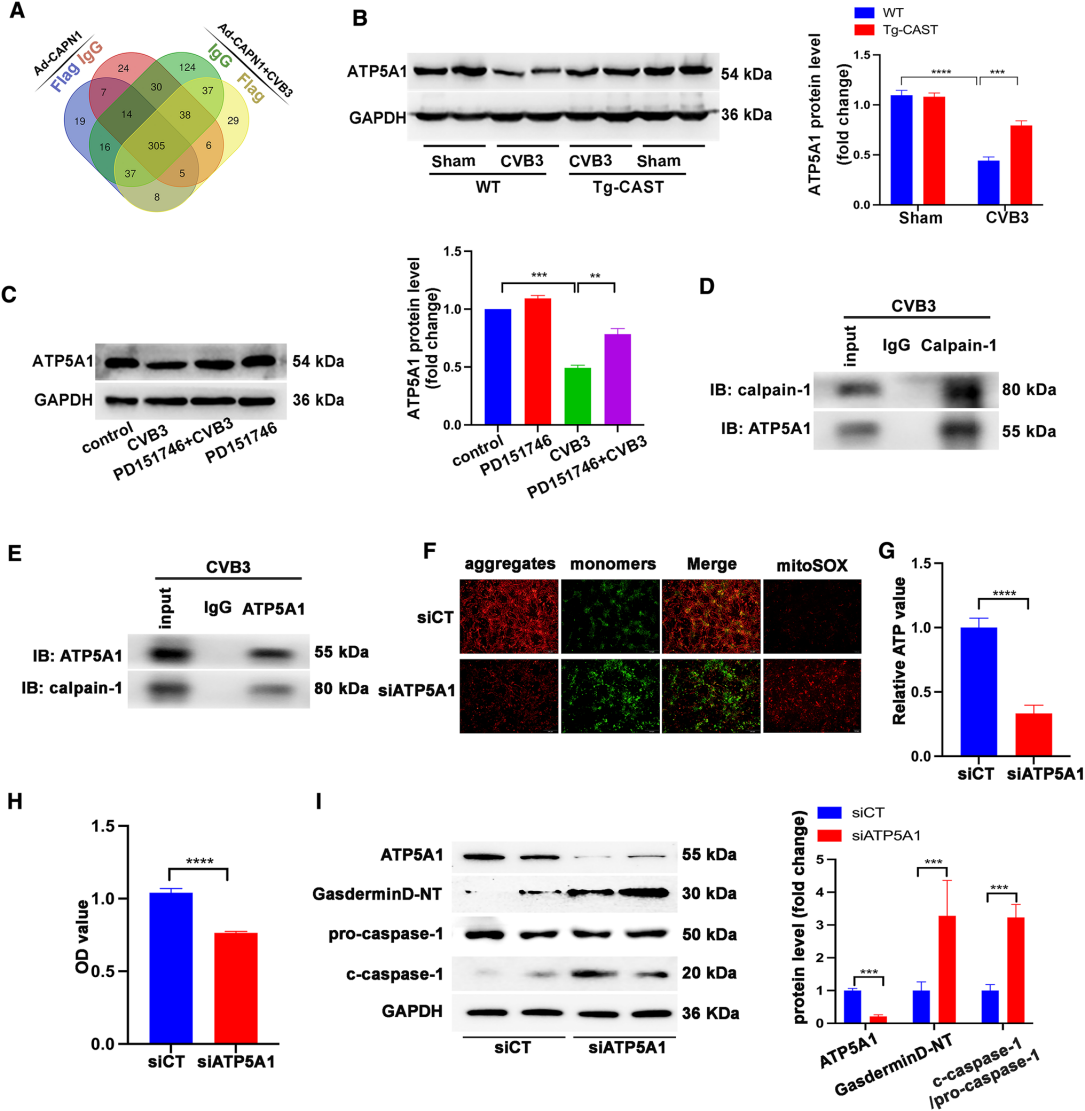
**A** Venn diagram showing eight proteins coexisting in NRCMs treated with Ad-CAPN1 or co-treated with Ad-CAPN1 and CVB3, as determined by co-immunoprecipitation using anti-IgG or anti-Flag antibody and subjected to mass spectrometry to determine calpain-1-interacting proteins. **B** Representative image of western blotting results and the quantitative analysis of ATP synthase-α (ATP5A1) in mice. *N* = 6 mice in each group. **C** Representative image of western blotting results and the quantitative analysis of ATP synthase-α (ATP5A1) in NRCMs subjected to the indicated treatments. *N* = 3 independent experiments. **D** Immunoprecipitation assay using anti-calpain-1 antibody or anti-IgG antibody (negative control antibody). Calpain-1 and ATP5A1 levels in co-immunoprecipitated samples. **E** Immunoprecipitation assay using anti-ATP5A1 antibody or anti-IgG antibody (negative control antibody). Calpain-1 and ATP5A1 levels in co-immunoprecipitated samples. **F** NRCMs were transfected with siRNA-Scramble (siCT) or siRNA-ATP5A1 (siATP5A1), and then, JC-1 staining indicated the mitochondrial membrane potential and mitoSOX indicated mitochondrial ROS. **G** ATP levels in NRCMs transfected with siCT or siATP5A1. *N* = 6 replicates from three independent experiments. **H** CCK8 assay showing the viability of NRCMs. *N* = 16 replicates from three independent experiments. **I** Representative image of western blotting results and the quantitative analysis of ATP5A1, gasdermin D-NT, pro-caspase-1, and c-caspase-1 in siCT- or siATP5A1-infected NRCMs. *N* = 3 independent experiments

To further explore the direct target of calpain-1 in cardiomyocytes under CVB3 infection, we overexpressed calpain-1 in neonatal rat cardiomyocytes with Ad-CAPN1, followed with or without CVB3 infection, and then, the extracted proteins were co-immunoprecipitated with anti-Flag antibody or control anti-IgG antibody and subjected to mass spectrometry. False-positive proteins, that is, the overlapping proteins between anti-Flag antibody-immunoprecipitated proteins and the corresponding controls, were screened out and eight proteins were identified to be immunoprecipitated by anti-Flag antibody in Ad-CAPN1-infected cells, with or without CVB3 treatment (Fig. 7A). Of these eight, ATP synthase-α (ATP5A1) is reported to be linked to ATP production, participating in mitochondrial function [37]. Therefore, we detected the expression of ATP5A1 in the WT + CVB3 and Tg-CAST + CVB3 groups. We found that CVB3 infection decreased ATP5A1 expression in WT mice but not in Tg-CAST mice (Fig. 7B), suggesting that activated calpain in response to CVB3 infection likely cleaves ATP5A1, leading to lower levels of functional ATP5A1 protein. Results from the in vitro experiment also showed that CVB3 infection decreased the levels of ATP5A1, while PD151746 co-treatment reversed this phenomenon (Fig. 7C). Furthermore, after treating NRCMs with CVB3, detectable ATP5A1 expression in anti-calpain-1 antibody-immunoprecipitated sample and detectable calpain-1 expression in anti-ATP5A1 antibody-immunoprecipitated sample corroborated that calpain-1 interacts with ATP5A1 in NRCMs (Fig. 7D, E). siATP5A1 was transfected into NRCMs to knock down ATP5A1 expression (Fig. 7I) and subsequent JC-1 staining and MitoSOX staining revealed that siATP5A1 decreased mitochondrial Δψm and increased mitochondrial ROS (Fig. 7F). ATP production was also reduced by ATP5A1 downregulation (Fig. 7G). In addition, compared to control siRNA, siATP5A1 reduced the viability of NRCMs (Fig. 7H), and promoted the activation of the NLRP3 inflammasome, as determined by the increased levels of gasdermin D-NT, and c-caspase-1 (Fig. 7I).

## Discussion

**Fig. 8:**
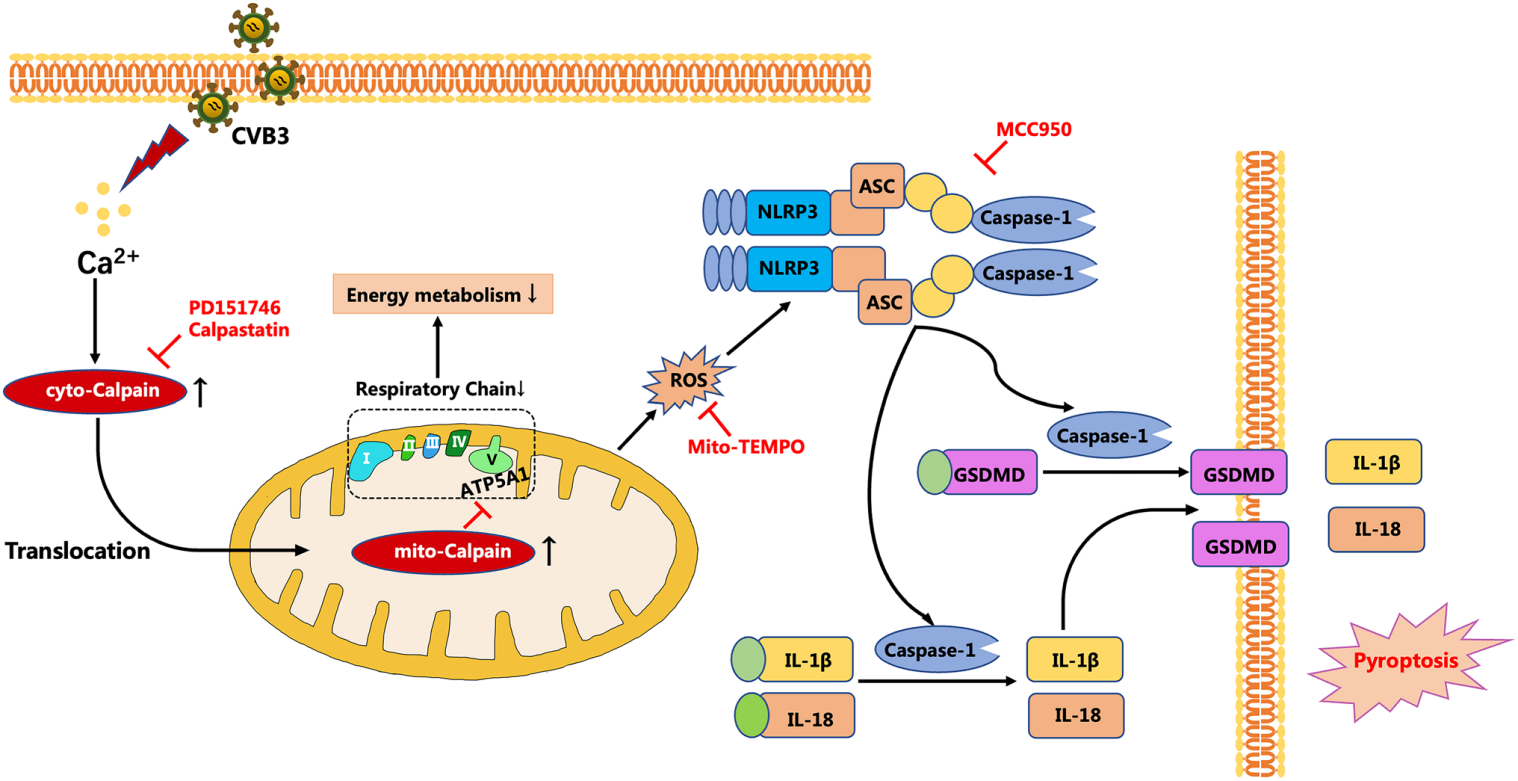
Calpain-1 activated under CVB3 stimulation promoted NLRP3 inflammasome-dependent pyroptosis through ATP5A1 cleavage and ROS overproduction in mitochondria

In this study, we demonstrated that calpain inhibition in vivo and in vitro alleviated CVB3-induced myocardial injuries by restraining the activation of the NLRP3 inflammasome. Furthermore, we established that mitochondrial function was altered by the effects of calpain-1 under CVB3 treatment due to calpain-1 translocation to mitochondria and cleavage of ATP5A1, leading to ATP synthesis deficiency, mitochondrial ROS overproduction, mitochondrial dysfunction, and subsequent activation of the NLRP3 inflammasome and pyroptosis (Fig. 8).

Mitochondria are cell organelles producing the majority of ATP by the means of oxidative phosphorylation; thus, mitochondria are not only a source of ATP energy but also generators of reactive oxygen species (ROS) that cause oxidative damage. Several studies propose a bidirectional link between mitochondrial malfunction, ROS, and chronic inflammatory diseases [49]. For example, mitochondrial dysfunctions have been unraveled in various autoimmune diseases, such as RA, SLE, and diabetes [9], while the expression of pro-inflammatory cytokines such as TNF-α, IL-6, and IL-1β is upregulated in the adipose tissues of obese and diabetic subjects [17]. Impressively, mitochondrion-derived DAMPs, such as mtDNA, can directly induce inflammatory changes in microglial and neuronal cells [58]. In addition, mitochondrial DNA that escapes from autophagy cell autonomously leads to Toll-like receptor (TLR) 9-mediated inflammatory responses in cardiomyocytes and can induce myocarditis and dilated cardiomyopathy [38], indicating that signals from mitochondria can modulate the inflammatory response. However, the causal relationship between mitochondrial dysfunction and disease induction or maintenance is still unclear. Implications of mitochondrial abnormity in viral myocarditis have also been reported. Previous studies showed that enterovirus replication in hearts induced mitochondrial apoptotic pathways in patients with acute myocarditis [54], and mitochondrial membrane phospholipid localization and mtDNA deletion rate were impaired in the myocardium after CVB3 infection [57]. Moreover, regulating mitochondrial function alleviated CVB3-induced myocarditis. Inhibiting mitochondrial permeability transition pore opening through cyclosporin A treatment reduced Abcc6-dependent cardiac necrosis and calcification following CVB3 infection in mice [35]. Furthermore, curtailing excessive mitochondrial fission with Dynamin-related protein 1 inhibitor rescued myocardial injury induced by CVB3 [44]. However, the precise molecular mechanisms underlying the association between mitochondria and myocarditis development need to be investigated further. In this study, we showed that mitochondrial abnormity appeared ahead of the inflammatory response during early CVB3 infection, indicating a potential causal relationship between CVB3-induced mitochondrial dysfunction and cardiac inflammatory injuries and that mitochondria acts as a mediator between activated calpain and cardiac injuries under CVB3 infection. Moreover, consistent with the previous conclusion that mitochondria integrate diverse signals and cause NLRP3 inflammasome activation leading to downstream inflammatory responses [53], the present study suggested that mitochondria dysfunction was an upstream signal to NLRP3 inflammasome activation in the context of CVB3-induced myocarditis. Thus, manipulating mitochondrial function during myocarditis might serve as a therapeutic target to improve cardiac function and inhibit the development of myocarditis to DCM.

Calpains are cytosolic calcium-activated cysteine proteases. Emerging data have suggested their participation in heart failure caused by hypertension, diabetes, atherosclerosis, ischemia reperfusion injury, atrial fibrillation, etc. The role of calpains in ventricular hypertrophy, inflammation, and fibrosis has also been demonstrated [27]. Thus, targeting the calpain pathway may be a novel therapeutic approach for patients with heart failure. Our previous studies showed that calpain activation induced by CVB3 infection facilitated virus replication, inflammation, and enhanced autophagy and apoptosis [29, 30], proposing that calpain may be a novel target for viral myocarditis treatment in clinical practice. However, as calpains function through cleaving a variety of substrates such as enzymes and structural and signaling proteins, identification of the mechanism involved is essential before using it as a therapeutic target. Although the mitochondria translocation of calpain-1 has been reported in other heart diseases, to the best of our knowledge, this is the first evidence showing that in myocarditis, calpain-1 translocates to mitochondria and then led to deficiency of mitochondrial ATP synthesis and excess production of ROS. We further established that the cleaved target of calpain-1 in mitochondria is ATP5A1. ATP5A1 downregulation resulted in NLRP3 inflammasome activation and subsequent pyroptotic injuries in cardiomyocytes, reinforcing the previous findings that membrane potential- and calpain-dependent reversal of caspase-1 inhibition activated canonical NLRP3 inflammasome [62].

Myocarditis is identified as an inflammatory disease of the heart muscle cells [45]. Initial activation of immune response in myocarditis is beneficial to the host by limiting viral spread; however, a persistent and excessive immune response conveys harmful consequences, contributing to the progression of myocarditis and DCM [14]. Thus, regulating the immune response can help migrate the detrimental effects of the host immune response and inhibit myocarditis development [31]. NLRP3 inflammasome is a critical component of the innate immune system. Aberrant activation of the NLRP3 inflammasome has been linked with several inflammatory disorders, such as cryopyrin-associated periodic syndromes, Alzheimer’s disease, diabetes, and atherosclerosis [23], as well as viral myocarditis, whereas the role of the NLRP3 inflammasome in viral myocarditis is controversial. Upon infection, various cardiac-resident cells, such as cardiomyocytes, endothelial cells, mast cells, phagocytes, and fibroblasts, secrete cytokines IL-1β, and IL-18 [7, 10], contributing to acute inflammation, and treatment with IL-1β could break the resistance of C57Bl/6 (H‐2b) mice to viral infection [43]. Moreover, the previous studies have shown that CVB3-induced myocardial NLRP3 contributed to the development of viral myocarditis [36, 55]. These results implicate the NLRP3 inflammasome in the development of myocarditis. However, another study showed that NLRP3 knockout mice manifested more severe cardiac and pancreatic lesions as well as worse cardiac dysfunction than WT control mice [56]. The discrepancy may be due to the different genetic backgrounds of mice. It must be noted that the knockout models were produced on a C57Bl/6 genetic background; however, the WT C57Bl/6 mice are relatively more resistant to CVB3 infection and fail to develop chronic disease. Moreover, in the NLRP3 knockout mice study, NLRP3 knockout was more associated with significantly increased virus loads, although chronic and excess inflammatory responses constitute vital contributors in the development of myocarditis. In our study, we demonstrated that activated calpain in viral myocarditis promoted the activation of the NLRP3 inflammasome and then exacerbated viral myocarditis; therefore, inhibiting NLRP3 inflammasome activation may be beneficial in viral myocarditis. Our study provides new perspectives on the regulation of NLRP3 inflammasome in myocarditis and suggests that calpain-1 can affect not only the expression of NLRP3 but also the assembly of the inflammasome complex. Therefore, targeting the calpain-1 pathway is a novel therapeutic approach for repressing the activation of the NLRP3 inflammasome for the treatment of myocarditis and other inflammatory diseases.

Some immunomodulatory drugs, including high-dose intravenous immunoglobulin, azathioprine, steroids, and cyclosporine A, target abnormally active immune cells in myocarditis [19]. Due to the diverse roles of immune cells involved in the pathogenesis of myocarditis, further investigation is required to identify accurate immunotherapies for special cell types. Targeting NLRP3 inflammasome with calpain-1 inhibitor has been shown to control myocarditis progression; however, the benefit of calpain inhibition has not been demonstrated in a clinical trial. There is a lack of approaches to inhibit the NLRP3 inflammasome, whereas IL-1β signaling can be blocked systematically using a competitive inhibitor of the IL-1β receptor (canakinumab) or blocking antibodies against IL-1β (anakinra) [20]. In clinical trials, canakinumab has been shown to be beneficial in the avoidance of post-myocardial infarction heart failure and in lowering the rate of recurrent cardiovascular events in atherosclerotic disease [1, 8, 41]. Anakinra has been considered standard care as a second-line treatment for patients with recurrent/refractory pericarditis as it improved outcomes in patients with pericarditis [2]. Importantly, anakinra treatment improved heart function in one case of fulminant myocarditis [3], but more clinical studies are needed. In addition, SGLT2 inhibitors have been reported to benefit heart failure patients through IL-1β and/or NLRP3 inflammasome mechanisms [24, 61], and may be used for immune modulation.

Our experiments were based on male Balb/c mice. As reported extensively, viral infection-induced myocarditis occurs more frequently in men than women, and male BALB/c mice infected with CVB3 develop more severe inflammatory heart disease compared to female mice [11]. Although the mechanisms are not fully elucidated, plenty of studies have reported the underlying factors contributing to this gender bias, including differences in pro-inflammatory cytokines, innate, and adaptive immune cells [12]. As a result, whether the function of calpain or activation of NLRP3 inflammasome or mitochondrial behavior is altered in different gender of mice, or whether they play roles in the biased susceptibility to CVB3 myocarditis needs to be determined further. It is known that the structural and functional parameters of the immune system in BALB/c and C57Bl/6 mice differ under physiological conditions, like that the humoral immune reactions mediated by type 2T helper cells (Th2) prevails in BALB/c mice, while C57Bl/6 mice are genetically predisposed to the predominance of cellular immunity (Th1) [60]. The baseline differences in immunological reactivity of BALB/c and C57Bl/6 mice contribute to various sensitivities of these animals to pathogenic agents, tumor growth, and autoimmune diseases [51]. As reported, BALB/c mice are susceptible strain to CVB3-induced myocarditis, whereas C57BL/6 mice showed the lowest susceptibility [5]. C57BL/6 mice possess hereditary resistance to viral cardiomyopathy, eliminating the virus following mild acute myocarditis, and no chronic inflammation would detect. Plenty of studies have investigated the underlying susceptibility factors that modulate the course of viral myocarditis [21, 26, 40]. Therefore, NLRP3 inflammasome or other receptor-mediated inflammasome might be affected by strain difference, which might contribute to the different morphofunctional characteristic of the immune system in BALB/c and C57Bl/6 mice.

In summary, our findings suggest the important role of calpain in the regulation of the NLRP3 inflammasome during the development of viral myocarditis. Calpain may be considered a potential therapeutic target for myocarditis treatment via inhibition of NLRP3 inflammasome activation through the mitochondrial pathway.
